# A model investigating environmental factors that play a role in female fecundity or birth rate

**DOI:** 10.1371/journal.pone.0207932

**Published:** 2018-11-27

**Authors:** Mary Regina Boland

**Affiliations:** 1 Department of Biostatistics, Epidemiology and Informatics, Perelman School of Medicine, University of Pennsylvania, Philadelphia, PA, United States of America; 2 Institute for Biomedical Informatics, University of Pennsylvania, Philadelphia, PA, United States of America; 3 Center for Excellence in Environmental Toxicology, University of Pennsylvania, Philadelphia, PA, United States of America; 4 Department of Biomedical and Health Informatics, Children’s Hospital of Philadelphia, Philadelphia, PA, United States of America; University of Missouri Columbia, UNITED STATES

## Abstract

**Objective:**

Over 12% of women in the United States have reduced fertility and/or fecundity. Environmental factors, such as temperature, and socioeconomic factors have been implicated in reducing female fecundity. The purpose of this study is to investigate the effect of environmental factors coupled with socioeconomic factors on birth rate at the country-level. We use birth rate as a proxy for female fecundity. This will enable us to identify the most important factors affecting female fecundity.

**Methods:**

Using country-specific data from **182 countries**, we constructed a regression model of the effects of environmental and socioeconomic factors on birth rate at the country-level. Our model assesses the role of temperature, Gross Domestic Product (GDP) per capita, fine air particulate matter (PM 2.5), and prevalence of male and female Body Mass Index (BMI) > = 25 (age-standardized) on birth rate per country. Because many of these factors are inter-dependent, we include all possible two-way interaction terms to assess the role of individual factors and interactions between multiple factors in the model.

**Results:**

In the full regression model, we found that GDP per capita along with 5 interaction terms were significant after adjusting for multiple testing. Female BMI was only nominally significant. GDP per capita was independently associated with birth rate (adjusted p-value <0.001). Prevalence of BMI > = 25 age-standardized in males and females were also significant when interacting with air pollution or GDP on female fecundity (birth rate). Temperature did not affect birth rate either independently or as an interaction unless BMI was removed from the model.

**Conclusion:**

A country’s economic wealth was the most significant factor in predicting birth rate in a statistical model that includes environmental and socioeconomic variables. This is important for future studies investigating environmental factors involved in increasing or decreasing female fecundity.

## Introduction

Over 12% of women in the United States have reduced fertility and/or fecundity [1]. Maternal birth season plays an important role in fertility in women [2–4]. Other fertility-related processes have been linked to birth season including the time of menarche [5] and menopause [6] in women. The main hypothesized biological mechanism underlying this relationship was that oocytes’ exposure to **high temperatures** at birth resulted in **increased oocyte loss** early in life [2, 7–9]. This reduction in oocyte volume thereby reduces a woman’s fertility when she goes to bear children later in life. This hypothesis was supported by the results from studies conducted in Austria [3], New Zealand [10] and NYC [2]. However, results from Vietnam [11] and Romania [12] appeared to find the opposite result, namely females born in high heat environments had ***increased*** fertility. This reveals an incomplete understanding regarding the relationship between seasonal temperature variation and female fertility/fecundity later in life.

Other factors are known to affect female fecundity and birth rate. High socioeconomic status (SES), which is related to income, is inversely correlated with fertility and birth rate [13, 14]. High-income countries provide their citizens with increased access to birth control methods and other forms of contraception[15]. Thereby affecting the birth rate. In addition fine air particulates (PM 2.5) or air pollution has been shown to affect the fertility rate [16] with a sperm related effect also reported [17]. On a global scale, air pollution is linked to poor countries (i.e., lower GDP) as poor countries often have more factories. This explains why countries with higher GDP often have improved environmental factors [18]. Finally, both male [19] and female [20] Body Mass Index (BMI) have been linked to birth rate and fertility outcomes through separate mechanisms. Furthermore, studies have stressed the importance of female waist-to-hip ratio in fertility with waist-to-hip ratio being more informative then BMI [21].

The purpose of this study is to investigate the relationship between environmental factors and socioeconomic factors on female fecundity as measured using birth rate. We aim to measure this at the country-level to simultaneously assess the effects of multiple socioeconomic and environmental factors from around the world on female fecundity (i.e., birth rate). We will also include interactions between the various inter-dependent factors to reveal the strongest global determinants in female fecundity.

## Materials and methods

### Integrating data from global datasets on birth rate and socioeconomic and environmental factors

We assembled a country-specific dataset on birth rate and other socioeconomic and environmental factors using data integrated from publically available data sources. For female fecundity, we used country-specific birth rate per 1,000 population as a proxy. To obtain birth rate data, we used data collected and maintained by the Central Intelligence Agency (CIA) [22]. For environmental factors, we obtained average annual temperature per country as measured in Celsius. This was obtained from the Climatic Research Unit [23]. We also obtained fine particulate matter (PM 2.5) from the World Health Organization (WHO). PM 2.5 data was obtained from the WHO’s Data Integration Model for Air Quality (DIMAQ). DIMAQ produces estimates of air pollution based on ground measurements and satellite retrievals of aerosol optical depth and chemical transport models [24]. The DIMAQ provides global annual exposures of PM 2.5 levels at high spatial resolution, which equates to approximately 11x11 kilometers at the equator. Data used in this paper is annual global estimates from 2014. For socioeconomic factors, we used the Gross Domestic Product (GDP) per Capita in 2016 from the WorldBank.org [25] to provide an overall assessment of a country’s wealth. We also included the prevalence of Body Mass Index (BMI) > = 25 (age-standardized) for males and females separately for each country. Each factor included in our analysis along with sources and agencies used to obtain the data are included in **Table 1.**

**Table 1:** Global data containing variables included in the fertility–temperature models.

Variable	Source/Agency Housing Data	Total Num. Countries Included in Dataset	Ref.
**Female Fecundity:**			
Birth rate per 1000 population	Central Intelligence Agency	226	[22]
**Environmental Factors:**			
Average annual temperature in Celsius from 1961–1990	Climatic Research Unit /Wikipedia	191	[23]
Fine Particulate Matter (PM 2.5)	World Health Organization	185	[24]
**Socioeconomic Factors:**			
Gross Domestic Product per Capita in 2016	WorldBank.org	264	[25]
Prevalence of Body Mass Index > = 25 (Age-Standardized) for Males and Females from 2014	World Health Organization	195	[26]

We included the total number of countries included in each dataset in **Table 1** to illustrate the variance across datasets. Determining the number of independent countries in the world is complex. There are 193 countries that are recognized as such by the United Nations (UN) with 2 countries–Vatican City and Palestine–having ‘permanent observer’ status at the UN [27]. In addition, 2 states–Kosovo and Taiwan–are only recognized by subsets of UN members [27]. According to the CIA World Factbook, and as of 2018, there are **195 recognized independent countries** [28]. We used these 195 countries as our definitive list of ‘countries’ with mappings available to the International Standards Organization (ISO) Alpha-3 standard country codes [28].

Clearly, some datasets contained many more country names (sometimes including non-independent states), especially the birth rate dataset and the Gross Domestic Product (GDP) dataset with 226 and 264 countries respectively. The GDP dataset also included assessments of regions that were given their own country codes, but were in fact groupings of other countries already included in the dataset. Therefore, we decided to integrate these datasets together, understanding that we would have at most only 185 countries captured in the integrated dataset because the air pollution dataset did not contain data from the all 195 independent countries. Whenever possible, we utilized the ISO Alpha-3 standard country codes to map across datasets. In some cases, this was not possible and we used the country names to map across datasets. However, different datasets used different nomenclatures for country-names. The birth rate and temperature datasets contained country names without corresponding ISO Alpha-3 codes. All records were manually reviewed to ensure that harmonization was accurate. Overall, our dataset contained information on six variables: birth rate, average air pollution, and average country temperature, GDP from 2016, BMI for males and BMI for females from 182 countries (**S1 Dataset**). This dataset captures 93.3% (182/195) of the independent states from around the world. Thirteen recognized independent countries by the CIA are missing from these analysis [28]. These thirteen countries are: Saint Kitts and Nevis, Liechtenstein, Monaco, Marshall Islands, Nauru, Palau, San Marino, South Sudan, Tonga, Tuvalu, Taiwan, Vatican City and Samoa. Our dataset of 182 countries is missing GDP information for 12 countries (Timor-Leste, Eritrea, North Korea, Djibouti, Papua New Guinea, Cuba, Syria, Iran, Libya, Venezuela, Andorra and Kuwait). and male and female BMI for 1 country (the Sudan).

### Regression model for relationship between environmental and socioeconomic factors and birth rate

We constructed a linear regression model with birth rate as the outcome variable. This is linear regression and therefore, we are predicting the actual birth rate per 1,000 individuals in the population and we did not use a binned birth rate statistic. We included in this full model, known predictors of fecundity (birth rate), including environmental factors: temperature and air pollution, socio-economic: GDP per capita and lifestyle: prevalence of BMI> = 25 (age-standardized) for males and females per country. We also included in this model pairwise interaction terms due to the inherent interdependency of these variables. A total of 16 terms were included in the model, including the intercept term, 5 covariates, and 10 interaction terms. We report the raw p-values, and also the Bonferroni-adjusted p-values (adjusted for 16 tests).

### Regression model for relationship between environmental and socioeconomic factors and birth rate without including body mass index

We also constructed a linear regression model excluding the two BMI factors. We again modeled birth rate as the outcome variable, and the following set of dependent variables: annual temperature, GDP, and fine air particulates (PM 2.5). In addition three pairwise interaction terms were included in the model (interaction of temperature and pollution; interaction of GDP and pollution and interaction of GDP and temperature). This model is the same as the one described in section 2.2 except that we removed the male and female BMI terms and their corresponding interaction terms.

## Results

### Global variance in birth rate and associated factors

**Fig 1:**
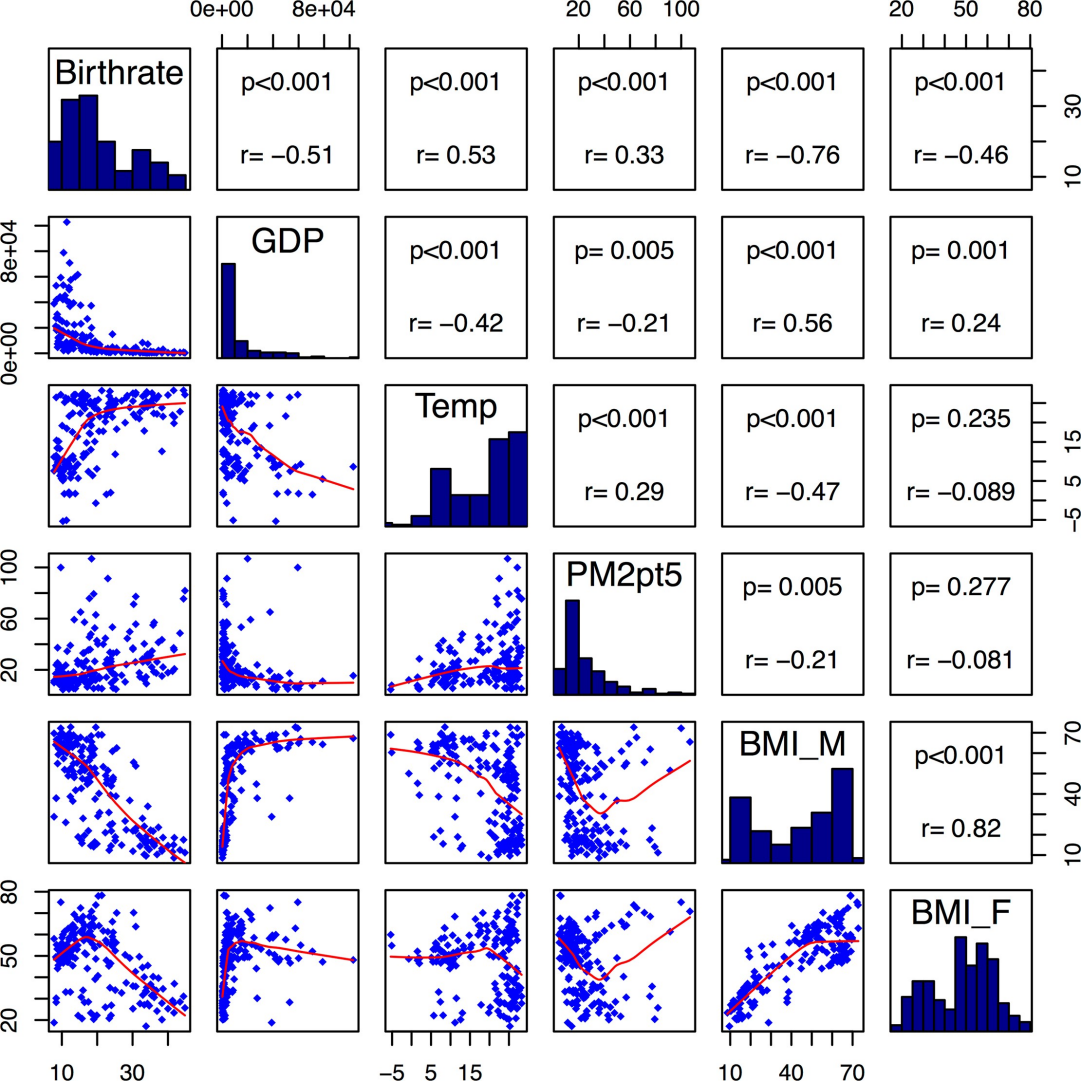
Correlation plots for birth rate and other factors known to affect birth rate including: Temperature, GDP, PM 2.5, BMI among Males (M) and females (F) On a global scale. Birth rate was correlated or anti-correlated with each factor known to play a role in female fecundity. 182 countries across the globe were included in this analysis. The red lines in the scatterplots located in the lower quadrant are lines derived using Locally Weighted Scatterplot Smoothing (LOWESS). These lines provide the overall shape of the distribution.

We investigated the relationship between fecundity (birth rate) and two environmental factors–temperature and air pollution–and also socio-economic factors–GDP, male and female BMI. We found that the prevalence of males with BMI > = 25 (age-standardized) was inversely correlated with fecundity across all countries (**Fig 1).** Interestingly, female BMI exhibited a different pattern. In countries with lower fecundity (between 10–20 births per 1,000 population), the prevalence of females with BMI> = 25 (age-standardized) was **positively correlated** with fecundity. However, when a country’s fecundity passed a certain threshold (around 20) then the prevalence of females with BMI > = 25 (age-standardized) became inversely correlated (**Fig 1).** Importantly, poor countries (low GDP) had higher air pollution and were warmer overall (**Fig 1**). This is an important consideration for analyses that demonstrate the effect of temperature on fecundity and was part of our motivation to include all factors into our global analysis of factors affecting female fecundity.

**Fig 2:**
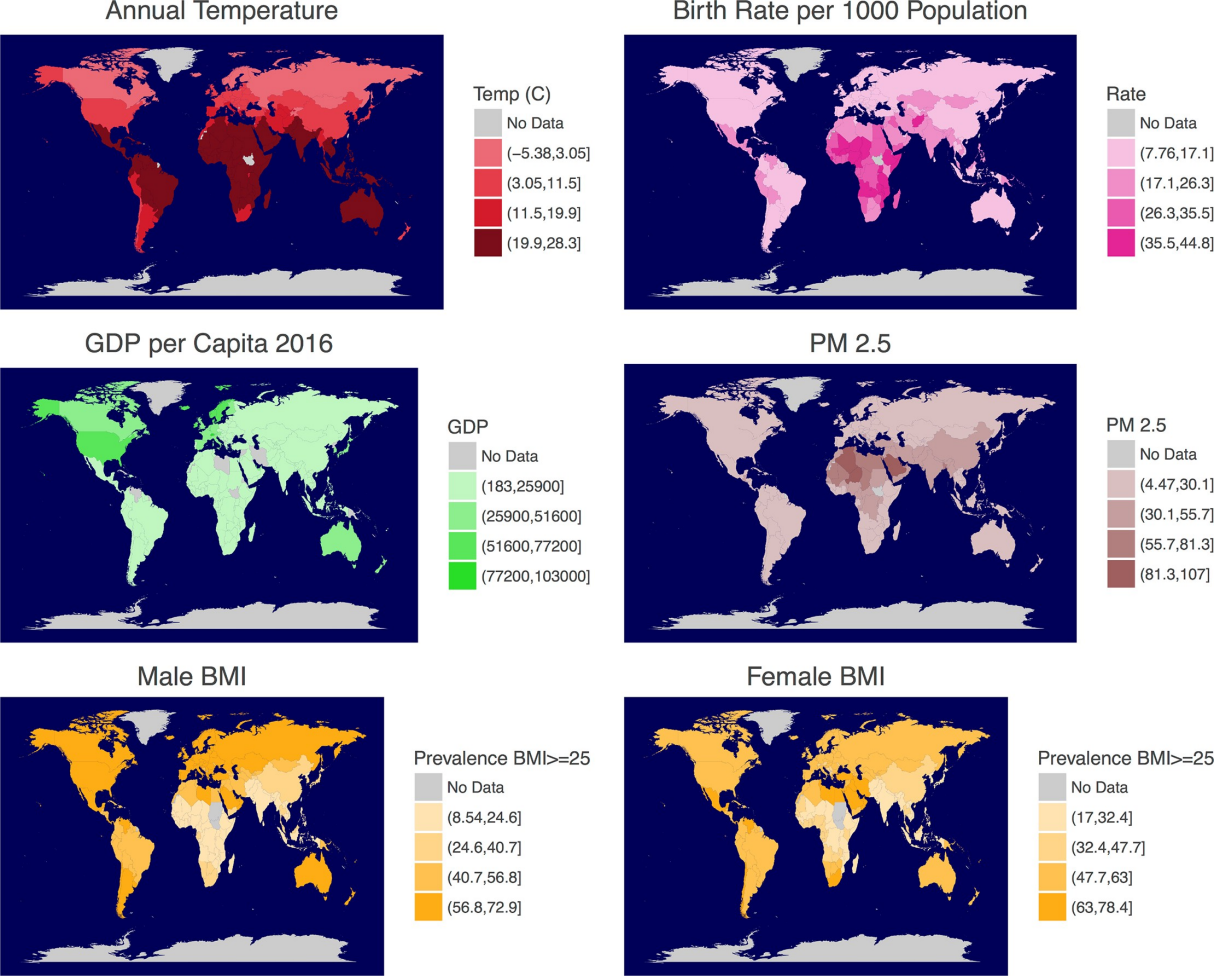
Global variation is evident in birth rate (i.e., fecundity), annual temperature, GDP, air pollution (PM 2.5) and body mass index (BMI) in both males and females. Many variables that affect fertility vary across the globe along with birth rate. Therefore, statistical models that explain this variation can help inform our understanding of the factors underlying the female fertility–birth season relationship.

The global variation in birth rate and its correlated factors can also be observed from the maps (**Fig 2**). Asia, South America and Africa have high temperatures and in general high fecundity (birth rate). However, these countries are also poorer (low GDP) and are exposed to higher air pollution levels (**Fig 2**). These countries also have lower prevalence of BMI > = 25 (age-standardized) for females and males. (**Fig 2**), which could be driven by the type of work they engage in (e.g., increased manual labor).

Many of the factors that are involved in fecundity are also correlated or anti-correlated with each other. Therefore, regression models that include interaction terms are important to capture this source of variation. **Fig 1** illustrates the correlation among the six factors. Birth rate on its own was significantly correlated with each variable. Therefore each was included in the regression model described in section 3.2. Birth rate was negative correlated with GDP (R = -0.51, p<0.001), positively correlated with temperature (R = 0.53, p<0.001), slightly positively correlated with air pollution (R = 0.33, p<0.001) and negatively correlated with male and female BMI (R = -0.76 and R = -0.46, respectively). Interestingly, male BMI was more strongly correlated with birth rate then female BMI. However, this is likely due to the interaction between GDP, pollution and BMI among males (often low-income males work in more labor intensive occupations). Hence our regression model (described in section 3.2) includes additional interaction terms to determine the most important factors.

### Regression model results for birth rate: Integrating data from across the globe

Our comprehensive model of environmental and socioeconomic factors and their role on female fecundity (birth rate) included average temperature per country from 1961–1990, GDP per capita, average air pollution (PM 2.5) and prevalence of BMI > = 25 (age-standardized) for males and females. We also included 10 pairwise interaction terms in the model. We report nominal p-values and also Bonferroni adjusted p-values (adjusting for 16 tests–one test for each factor included in the model). The model results are shown in **Table 2.**

**Table 2:** Regression model with BMI Included as confounder.

Variable Included in Model	Estimate	CI 2.5%	CI 97.5%	P-value	Adjusted P-value
**Covariates**					
Average Temperature 1961–1990 (Celsius)	-0.11	-0.81	0.60	0.77	1
GDP per Capita in 2016 USD	-8.02 X 10^−4^	-0.0012	-0.0005	**1.18 X 10**^**−5**^	**1.88 X 10**^**−4**^
Annual Estimated Average PM 2.5 in 2014	-0.30	-0.604	0.008	0.06	0.90
Prevalence Male BMI > = 25 (age-standardized) in 2014	-0.33	-0.71	0.042	0.08	1
Prevalence Female BMI > = 25 (age-standardized) in 2014	-0.47	-0.92	-0.02	**0.04**	0.65
**Interaction Terms** **(term 1 : term 2)**					
Prevalence Male BMI > = 25 (age-standardized) in 2014: Prevalence Female BMI > = 25 (age-standardized) in 2014	6.51 X 10^−3^	2.26 X 10^−3^	0.01	**0.003**	**0.046**
Temperature in C: GDP per Capita in 2016 USD	9.91 X 10^−6^	-3.98 X 10^−7^	2.02 X 10^−5^	0.06	0.95
Temperature in C: Annual Estimated PM 2.5 in 2014	3.30 X 10^−3^	-9.00 X 10^−3^	0.016	0.60	1
Temperature in C: Prevalence Male BMI > = 25 (age-standardized) in 2014	-0.015	-0.032	0.001	0.07	1
Temperature in C: Prevalence Female BMI > = 25 (age-standardized) in 2014	0.015	-0.004	0.034	0.13	1
GDP per Capita in 2016 USD : Prevalence Female BMI > = 25 (age-standardized) in 2014	-1.57 X 10^−5^	-2.47 X 10^−5^	-6.64 X 10^−6^	**7.84 X 10**^**−4**^	**0.013**
Annual Estimated PM 2.5 in 2014: Prevalence Female BMI > = 25 (age-standardized) in 2014	0.019	0.009	0.029	**2.89 X 10**^**−4**^	**0.005**
GDP per Capita in 2016 USD: Prevalence Male BMI > = 25 (age-standardized) in 2014	2.29 X 10^−5^	1.41 X 10^−5^	3.17 X 10^−5^	**8.68 X 10**^**−7**^	**1.39 X 10**^**−5**^
Annual Estimated PM 2.5 in 2014: Prevalence Male BMI > = 25 (age-standardized) in 2014	-0.016	-0.026	-0.006	**0.002**	**0.038**
GDP per Capita in 2016 USD: Annual Estimated PM 2.5 in 2014	-1.75 X 10^−6^	-6.31 X 10^−6^	2.80 X 10^−6^	0.45	1

P-values are **bold** if they are significant (<0.05), and rows containing significant terms are shown in grey

Both female BMI and GDP per capita were nominally significant (p-value <0.05) in the model for birth rate. Although prevalence of female BMI > = 25 was not significant after adjusting for multiple testing (**Table 2**). After adjusting for multiple testing, GDP per capita was still significantly negatively correlated with birth rate (p<0.001). Five interaction terms were also significant.

Significant interaction terms included an interaction between male and female BMI > = 25 (age-standardized); GDP and BMI (male and female each a separate term); and air pollution and BMI. The air pollution (PM 2.5) term was not associated with birth rate in the full model (unadjusted p = 0.06, and adjusted p = 0.90), but only interactions between air pollution and BMI. Overall, prevalence of female BMI > = 25 (age-standardized) was nominally significant in determining birth rate independently and also via indirect mechanisms (i.e., GDP, air pollution, male BMI); whereas, prevalence of male BMI > = 25 (age-standardized) was not independently associated with birth rate. This indicates that the strong anti-correlation between prevalence of male BMI > = 25 (age-standardized) and female fecundity is through correlation with other variables (**Fig 1**).

### Regression model predicting birth rate without body mass index

The model result for our linear regression model constructed without the two BMI terms is shown in **Table 3.** This model is the same as the one described in section 2.2 except that we removed the male and female BMI terms and their corresponding interaction terms. We used this second model to determine how the inclusion of BMI affects the environmental factors relationship with fecundity (birth rate). Temperature and the interaction term for temperature and GDP were significant. Importantly, GDP was not significant in this model, but only the interaction between GDP and temperature.

**Table 3:** Regression model results for factors that influence birth rate without including BMI.

Variable Included in Model	Estimate	CI 2.5%	CI 97.5%	P-value	Adjusted P-value
**Covariates**					
Average Temperature 1961–1990	0.65	0.205	0.819	**0.001**	**0.009**
GDP per Capita in 2016	7.26 X 10^−5^	-5.07 X 10^−5^	1.96 X 10^−4^	0.246	1
PM 2.5	-0.038	-0.307	0.232	0.781	1
**Interaction Terms** **(term 1 : term 2)**					
Temperature: GDP	-2.05 X 10^−5^	-3.01 X 10^−5^	-1.10 X 10^−5^	**3.40 X 10**^**−5**^	**2.38 X 10**^**−4**^
Temperature: PM 2.5	0.007	-0.004	0.019	0.195	1
GDP: PM 2.5	-1.50 X 10^−6^	-5.13 X 10^−6^	2.14 X 10^−6^	0.417	1

P-values are **bold** if they are significant (<0.05), and rows containing significant terms are shown in grey

Without BMI in the model, temperature and the interaction of temperature and GDP were significantly associated with birth rate. Importantly temperature was positive associated with fecundity (estimate = 0.65, adjusted p-value = 0.009). The interaction between temperature and GDP was significant with adjusted p-value = **2.38 X 10**^**−4**^ (**Table 3**). None of the air pollution terms in this model were significant.

## Discussion

In this study, we constructed a country-level model of factors involved in fecundity (birth rate) using data from 182 countries across the world. A high-level global analysis was performed to look at factors correlated with birth rate on the global scale to identify the most important factors in female fecundity or birth rate. We modeled interactions between multiple factors because of the high-degree of interdependency among factors. Overall, we found that a country’s economic wealth (i.e., GDP per capita) was the most significant in predicting fecundity in a full model that includes male and female BMI (**Table 2**). Air pollution (PM 2.5) and BMI were also important contributory factors (as interaction terms) in predicting fecundity (birth rate). However, temperature did not affect birth rate either independently or as an interaction unless the BMI terms were removed from the model (i.e., prevalence of BMI > = 25 in males and females, age-standardized).

Many studies have pointed to the role of temperature on female fecundity. Some studies point to an in-direct mechanism, through impairment of the male’s fertility. Spermatogenesis in males is impaired when temperature increases resulting in impaired fertility [29]. This suggests that certain seasons of the year may result in increased fertility periods due to increases in sperm viability and quality [30]. Male sperm motility and/or concentration (but not quality) have been linked to seasonally dependent variables including vitamin D [31] and vitamin C (ascorbic acid) [32]. Many of these seasonally dependent variables (e.g., vitamin D) are also related to temperature variation in different regions of the world. However, the results of our model point to the role of income (GDP) on female fecundity and interactions between GDP and BMI, air pollution (PM 2.5) and GDP on female fecundity. Temperature was insignificant in the model with BMI (**Table 2**), but was significant in the model without BMI (**Table 3**). Studies in insects have shown that temperature and the size of the female were both important in determining fecundity indicating that temperature was not the only variable of importance [33]. Studies in humans have shown that body fat distribution was more important in fertility outcomes then age or obesity status [34] indicating the importance not only of BMI but also body fat distribution on fecundity.

Importantly, we include the results of the model without the inclusion of the male and female BMI parameters (i.e., prevalence of BMI> = 25, age-standardized) in **Table 3.** In the model without BMI, temperature was a significant factor even after adjusting for multiple testing (p = 0.009). Temperature is highly correlated with many other variables that are important in fecundity, which was the rationale for constructing this global model. In addition, the significant interaction terms point to the importance of capturing the inherent inter-dependency among a number of these variables (**Fig 1).** In addition, the significant interaction term may point to a third un-measured variable that is underlying both factors that could explain the fecundity effect.

Significant factors in the full model (**Table 2)** demonstrate the importance of other factors (e.g., BMI) and their interactions with environmental factors (BMI and air pollution) on female fertility and birth rate on a global scale. This is important because many of these factors correlate with temperature indicating that earlier work seeking to demonstrate a relationship between perinatal exposure of females to high temperatures decreases female fertility later in life may be capturing an environmental exposure that is coupled with temperature, but not temperature itself [2, 3, 10–12].

Importantly, our analysis focuses on birth rate, which is a measure of fecundity. We are not able to assess the number of pregnancies lost in various countries and how variance in pregnancy loss (i.e., spontaneous abortion or miscarriage) affects a country’s birth rate. Pregnancy loss is very difficult to capture in many countries due to lack of reporting. Even outcomes such as preterm birth and still birth (which are easier to capture then pregnancy loss) are still difficult to capture globally [35]. This is why we restricted our analyses to birthrate. Temperature has been implicated in pregnancy loss and conception rates in several studies among dairy cows [36–39]. We have found that temperature was only significantly related to a country’s birth rate if BMI was excluded (**Table 3**). However, in a full model that includes male and female BMI, the GDP of the country was the most significant variable along with interactions between air pollution, BMI and GDP (**Table 2**). Some of these findings could be related to our inability to assess the pregnancy loss rate across countries.

There are several limitations to this study. First, we perform a global analysis investigating each country equally. However, not all countries are the same size. We use statistics such as birth rate per 1,000 population and GDP per capita, which should help to address some of these issues. However, there are some fundamental differences between large and small countries that are not fully addressed by our methods. Therefore, this remains a limitation of our approach. We only investigated variables that have been reported to impact female fecundity and that had data available at the country-level for the majority of countries included in our study. Therefore, not every factor implicated in female fecundity was included in our model. The major factors (income, body mass, temperature and air pollution) were included.

## Conclusions

In conclusion, we constructed a global model of female fecundity using data from 182 countries. Our model captured known environmental and socioeconomic factors that have been implicated in reducing or increasing female fecundity. We included at the country-level measures of average annual temperature, average air pollution, GDP per capita and prevalence of BMI > = 25 for males and females (age-standardized). We also included interaction terms to capture the inherent inter-dependency among many of these factors. We found that GDP per capita was independently associated with fecundity (birth rate). In addition, air pollution (PM 2.5), and BMI were important contributory factors as interaction terms. BMI was found in significant interactions with GDP and air pollution. Annual temperature was only significant in the model that did **not** include BMI pointing to other mechanisms underlying the temperature to fecundity relationship.

### Ethics approval and consent to participate

This study only includes publically available data. No patient-level data was used only aggregate statistics therefore no consent was required to conduct this study.

## Supporting information

S1 Dataset Global fertility model dataset. All data included in the global fertility model for 182 countries is provided in this file.
(CSV)
